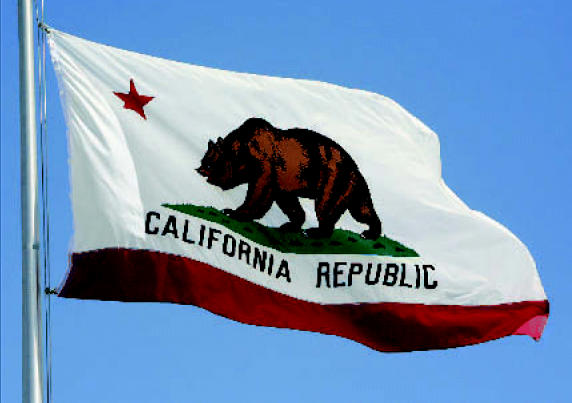# EHPnet: California Environmental Protection Agency

**Published:** 2007-03

**Authors:** Erin E. Dooley

Sizewise, California is the third largest state in the United States, it has the largest population, and its economy ranks among the top ten in the world. Because of its economic clout, laws that are made in the state can have a ripple effect throughout the country and even the world. One area of law in which California is making an impact is environmental issues. The California Environmental Protection Agency (Cal/EPA) website at **http://www.calepa.ca.gov/** provides information on the state’s many initiatives and programs.

At the center of the homepage is a Topics of Interest section, currently headed by information on the Cal/EPA Climate Action Team. This team was established by a June 2005 executive order signed by governor Arnold Schwarzenegger that also created greenhouse gas targets for the state. The team, which submitted its first biannual report to Schwarzenegger and the state legislature in April 2006, is composed of members from several state agencies and charged with implementing and monitoring programs for reducing emissions that contribute to global warming. The Climate Action Team section of the site contains the 2006 report, public comments on the draft of the report, and fact sheets on California’s climate change activities and policies.

Four of the nation’s busiest 20 ports are in California. The concentration of diesel emissions in these areas, where ships, trucks, and trains converge, contributes to a toxic mix of air pollutants that threatens the health of nearby residents. According to a September 2005 article in the *Los Angeles Times*, the port complex in that city has become the single largest air polluter in the Los Angeles Basin. The Cal/EPA Topics of Interest section has a link to the state’s recently unveiled Goods Movement Action Plan, which includes approximately 200 potential projects in areas including public health and environmental impact mitigation and community impact mitigation.

Another Topic of Interest centers on California’s efforts to develop hydrogen as an alternative fuel. Within the Hydrogen Highway Initiative section is information on pertinent laws passed by the state. The latest of these, Senate Bill 76, provides funding for state-funded hydrogen demonstration projects including fueling stations and the purchase of hydrogen-fueled vehicles. Also available are fact sheets, brochures, and other documents about these fuels.

Waste disposal is the fourth Topic of Interest currently featured on the website. According to the Cal/EPA, Californians have cut their amount of trash in half since 1989. Among other initiatives that have facilitated this progress is the California Take-It-Back Partnership, a project between the state government and the business sector to provide convenient drop-off points for toxic trash such as used batteries, fluorescent lamps, and electronic devices. Also in this section are pages for consumers that answer the questions of why, what, how, and where they can recycle, what “zero waste” is, and where all of California’s trash goes.

The Cal/EPA homepage also offers links to information on children’s environmental health, environmental justice, environmental sustainability, and the Education and the Environment initiative, which mandates a broad-ranging strategy to bring education about the environment into the state’s K–12 schools.

## Figures and Tables

**Figure:**